# Long-term efficacy and safety of fingolimod in Japanese patients with relapsing multiple sclerosis: 3-year results of the phase 2 extension study

**DOI:** 10.1186/s12883-017-0794-5

**Published:** 2017-01-28

**Authors:** Takahiko Saida, Yasuto Itoyama, Seiji Kikuchi, Qi Hao, Takayoshi Kurosawa, Kengo Ueda, Lixin Zhang Auberson, Isao Tsumiyama, Kazuo Nagato, Jun-ichi Kira

**Affiliations:** 1 Institute of Multiple Sclerosis Therapeutics, Nishinokyo-Kasugacho 16-44-409, Nakakyo-ku, Kyoto, 604-8453 Japan; 2 Kyoto Min-Iren-Central Hospital, Kyoto, Japan; 3 Kyoto University Hospital, Kyoto, Japan; 4 International University of Health and Welfare, 1-7-4 Momochihama, Sawara, Fukuoka City, Fukuoka 814-0001 Japan; 5 Hokkaido Medical Center, National Hospital Organization, 1-1 Yamanote 5-jo 7-chome, Sapporo, 063-0005 Japan; 6 Novartis Pharma KK, 1-23-1, Toranomon, Minato-ku, Tokyo, 105-6333 Japan; 7 Novartis Pharma AG, Fabrikstrasse 12, 4002 Basel, Switzerland; 8 Mitsubishi Tanabe Pharma Corporation, 17-10, Nihonbashi-Koamicho, Chuo-ku Tokyo, 103-8405 Japan; 9 Department of Neurology, Neurological Institute, Graduate School of Medical Sciences, Kyushu University, 3-1-1 Maidashi, Higashi-ku, Fukuoka, 812-8582 Japan

**Keywords:** Fingolimod, Multiple sclerosis, Phase 2 study extension, Relapse, Aquaporin 4

## Abstract

**Background:**

The low level of disease activity and manageable safety profile seen with fingolimod versus placebo in a 6-month, phase 2, randomized controlled trial in Japanese patients with relapsing multiple sclerosis (MS; ClinicalTrials.gov Identifier NCT00537082) were maintained in the initial 6-month observational study extension. Here, we report long-term safety and efficacy results of the 3-year follow-up to the phase 2 study extension.

**Methods:**

The 6-month core study was completed by 147 patients, of whom 143 entered the extension and took at least one dose of fingolimod. Those originally randomized to placebo were re-randomized to fingolimod 1.25 mg (*n* = 23) or 0.5 mg (*n* = 27). During the extension, the patients taking fingolimod 1.25 mg (*n* = 46) were switched to open-label fingolimod 0.5 mg, and those originally randomized to fingolimod 0.5 mg (*n* = 47) continued with open-label fingolimod 0.5 mg.

**Results:**

Continuous fingolimod treatment was associated with a sustained low level of MRI and relapse activity for the duration of the extension phase; 75–100% (range across all assessment time points up to end of study) of patients remained free of Gd-enhanced T1 lesions, 88–100% remained free of new/newly enlarged T2 lesions, and 45–62% remained relapse-free. In patients who switched to the active treatment, a 79.5% decrease in annualized relapse rate (ARR; from 1.131 before switch to 0.232 6-months after switch) was observed in the first 6 months of the extension phase and thereafter remained low until the end of study (0.16–0.31 across all assessment time points after switch up to end of study). The mean number of Gd-enhanced T1 and new/newly enlarged T2 lesions decreased up to month 9 and thereafter remained low until the end of study (0.0–0.1 and 0.0–0.3, respectively, across all assessment time points after switch up to end of study). Fingolimod was generally well-tolerated and the safety profile was consistent with the core and 6-month extension. Serious adverse events were reported in 13.3% of patients during the extension study, with the range in the continuous fingolimod and placebo-fingolimod switch groups (3.7–21.7%) being similar to that reported in the core study for the placebo and fingolimod groups (5.3–20.4%).

**Conclusion:**

Continuous fingolimod treatment over 36 months was associated with maintained efficacy and a manageable safety profile with no new safety signals. These results indicate that fingolimod provides long-term treatment benefit for Japanese patients with relapsing MS.

**Trial registration:**

ClinicalTrials.gov NCT00670449 (April 28, 2008).

**Electronic supplementary material:**

The online version of this article (doi:10.1186/s12883-017-0794-5) contains supplementary material, which is available to authorized users.

## Background

Fingolimod is a once-daily, orally administered therapy for relapsing forms of multiple sclerosis (MS). Currently, it is estimated that fingolimod has been used to treat approximately 155,000 patients in both clinical trials and post-marketing settings, with a total exposure of approximately 343,000 patient-years [1]. In the phase 2 and 3 trials, conducted in predominantly Caucasian populations, fingolimod treatment led to a significant reduction in clinical and magnetic resonance imaging (MRI) measures of disease activity compared with placebo [2–4] or interferon (IFN) beta-1a [5]. Similarly, a 6-month, phase 2, randomized controlled trial in Japanese patients with relapsing MS (ClinicalTrials.gov Identifier NCT00537082) demonstrated that fingolimod 0.5 and 1.25 mg significantly reduced relapse rates and MRI disease activity compared with placebo [6]. In the 6-month observational extension of this study, continuous fingolimod treatment for up to 12 months was associated with maintained or improved efficacy and a manageable safety profile [7]. The extension was continued until fingolimod received marketing authorization in Japan (28 November 2011). Here, we report long-term safety and efficacy results of the 3-year follow-up to the phase 2 extension study of fingolimod in Japanese patients with relapsing MS.

## Methods

### Study design and patient population

This was an extension of a 6-month, randomized, double-blind study comparing fingolimod 0.5 or 1.25 mg with placebo [6], conducted at 43 centers in Japan between 2008 and 2012. The study design, inclusion criteria and patient population have previously been described [6, 7]. Patients diagnosed with relapsing MS according to the McDonald criteria [8] were eligible to enter the study, and were randomized to fingolimod 0.5 or 1.25 mg, or placebo in a 1:1:1 ratio. Patients with longitudinally extensive spinal cord lesions of at least three vertebral segments (a marker of neuromyelitis optica [NMO]) detected by MRI at screening were excluded. Those who completed the 6-month core study had an option to enter the extension study. In the extension phase, patients originally randomized to placebo were re-randomized to dose-blind fingolimod 0.5 or 1.25 mg, and patients already treated with fingolimod continued on the same dose. Subsequent review of safety and efficacy data from the phase 3 clinical trials [4] revealed a more favorable benefit-risk profile using fingolimod 0.5 than 1.25 mg. Consequently, all patients treated with fingolimod 1.25 mg were switched to this lower dose by 22 February 2010, and the study adopted an open-label design until the end of the study.

### Efficacy and safety endpoints

Although no primary efficacy analysis was undertaken owing to the nature of the extension study, several efficacy and safety outcomes were assessed. MRI measures, including the number of lesions (both Gd-enhanced T1 and new or newly enlarged T2) by visit and treatment, and the proportion of patients free from these lesions were assessed at screening and months 3, 6, 9, 12, 18, 24 and 36. ARR and Expanded Disability Status Scale (EDSS) measurements were conducted every 3 months from month 6 onwards. Relapse activity (proportions of patients without relapses, time to the first confirmed relapse and ARR) was determined up to the end of the study. ARR was calculated using the total number of relapses experienced during a specific period of time adjusted to a 1-year period. Confirmed relapse was defined as new, worsening, or recurrent neurological symptoms that occurred at least 30 days after the onset of a preceding relapse, lasted at least 24 h without fever or infection and were accompanied by an increase of at least half a point on the EDSS or an increase of at least one point in two functional systems scores or of at least two points in one functional system score (excluding changes in bowel or bladder function and cognition). Confirmed disability was measured using the EDSS score; 3-month and 6-month confirmed disability progression was defined as a 3-month and 6-month sustained increase from core baseline in the EDSS score, respectively, with progression defined as a 1-point increase from baseline in patients with a baseline EDSS score of 0–5.0, or a 0.5-point increase in patients with a baseline EDSS score of 5.5 or above. All adverse events (AEs) and serious adverse events (SAEs) were summarized by patient group, and pregnancy outcomes were tracked. Regular monitoring of laboratory values and assessments of vital signs, electrocardiograms, physical condition and body weight were undertaken during patient visits. Additional safety assessments as specified per protocol included dermatological examinations, ophthalmic examinations, chest radiography and pulmonary function tests. Anti-aquaporin-4 (AQP4) antibody test results were collected retrospectively from medical records of patients who consented to provide the data during the extension study.

### Statistical analyses

All patients who were randomized in the 6-month core study and received at least one dose of study drug were analyzed as the core full analysis set (FAS) in the extension study. Efficacy analyses were performed on the core FAS; all analyses were based on descriptive statistics and no inferential analysis was performed. ARR was calculated by treatment group in the core FAS. A Kaplan-Meier analysis of time to first confirmed relapse from core baseline to end of study estimated the proportion of relapse-free patients at each time point. Data for patients with no confirmed relapse during this time and for those who dropped out or otherwise stopped treatment before a confirmed relapse occurred were all censored (i.e. the time to first onset of the confirmed relapse for these patients was set as the time in study from month 0 to the end of the study). No data imputation for drop-outs or missing values was used in this analysis.

Safety analyses were performed on the extension safety set, which included all patients who received at least one dose of the study drug in the extension period.

## Results

### Patient characteristics and fingolimod exposure

**Fig. 1:**
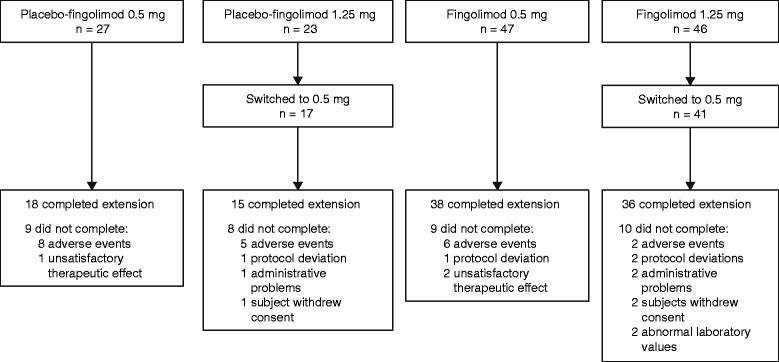
Enrollment and follow-up of patients who completed the core 6-month study and entered the long-term extension. A patient was defined as having completed the extension study if he/she was taking part in the study at the time of fingolimod launch in Japan

Of 147 patients who completed the core study, 143 entered the extension study (Fig. 1). Baseline characteristics were similar across treatment groups (placebo-fingolimod 0.5 mg; placebo-fingolimod 1.25 mg; fingolimod 0.5 mg, fingolimod 1.25 mg) (Table 1); approximately two-thirds (67.8%) of patients were female and the mean age was 35.1 years. The extension study was completed by 107 (74.8%) patients and 36 (25.2%) discontinued treatment (Fig. 1). AEs were the most common reason for treatment discontinuation (*n* = 21, [14.7%]), followed by protocol deviations (*n* = 4, [2.8%]), withdrawal of consent (*n* = 3, [2.1%]), administrative problems (*n* = 3, [2.1%]), unsatisfactory therapeutic effect (*n* = 3, [2.1%]) and abnormal laboratory values (*n* = 2, [1.4%]). In patients who received fingolimod 0.5 or 1.25 mg during the core study, and continued with treatment during the extension, the median duration of fingolimod exposure was 1180 and 1070 days, respectively. Patients who received placebo during the core study and then switched to fingolimod 0.5 or 1.25 mg at month 6 had a median duration of fingolimod exposure of 857 and 820 days, respectively (Additional file 1: Table S1).

**Table 1:** Baseline demographics and clinical characteristics of patients at entry to the core study (extension randomized population)

Characteristic	Placebo-fingolimod 0.5 mg (*n* = 27)	Placebo-fingolimod 1.25 mg (*n* = 23)	Fingolimod 0.5 mg (*n* = 47)	Fingolimod 1.25 mg (*n* = 46)
Age, years				
Mean (SD)	34.2 (9.1)	35.5 (8.4)	34.9 (9.0)	35.7 (8.8)
Median (range)	34.0 (18–52)	34.0 (21–51)	34.0 (19–52)	36.0 (18–55)
Female, *n* (%)	19 (70.4)	14 (60.9)	33 (70.2)	31 (67.4)
BMI, kg/m^2^				
Mean (SD)	21.0 (2.6)	20.7 (3.1)	21.8 (3.2)	22.0 (3.9)
Median (range)	20.8 (15.0–26.2)	20.2 (13.8–28.8)	21.5 (15.1–32.6)	21.1 (18.1–36.2)
Duration of MS since first symptom, years				
Mean (SD)	8.4 (8.1)	8.4 (7.2)	8.2 (6.6)	7.6 (5.5)
Median (range)	5.4 (1–27)	5.9 (1–24)	6.4 (1–26)	6.2 (0–21)
Number of relapses within previous year				
Mean (SD)	2.1 (2.1)	1.4 (0.7)	1.4 (0.9)	1.5 (1.0)
Median (range)	2.0 (1–12)	1.0 (0–3)	1.0 (0–3)	1.0 (0–4)
EDSS score				
Mean (SD)	1.9 (1.6)	2.4 (1.6)	2.4 (1.9)	1.9 (1.7)
Median (range)	1.5 (0.0–5.0)	2.0 (0.0–5.5)	2.0 (0.0–6.0)	2.0 (0.0–6.0)
Patients free of Gd-enhancing lesions				
*n* (%)	13 (48.1)	17 (73.9)	28 (59.6)	22 (47.8)
Number of Gd-enhancing lesions				
Mean (SD)	1.7 (2.5)	0.7 (1.5)	1.0 (1.6)	1.7 (2.42)
Median (range)	1.0 (0–9)	0.0 (0–5)	0.0 (0–5)	1.0 (0–9)
Number of T2 lesions				
Mean (SD)	28.9 (23.2)	33.3 (23.1)	30.3 (22.8)	34.6 (24.2)
Median (range)	23.0 (3–98)	35.0 (1–91)	24.0 (4–100)	29.5 (5–119)

*Abbreviations*: *BMI* body mass index, *EDSS* Expanded Disability Status Scale, *Gd* gadoliunium, *MS* multiple sclerosis, *SD* standard deviation

### Efficacy evaluation

**Fig. 2:**
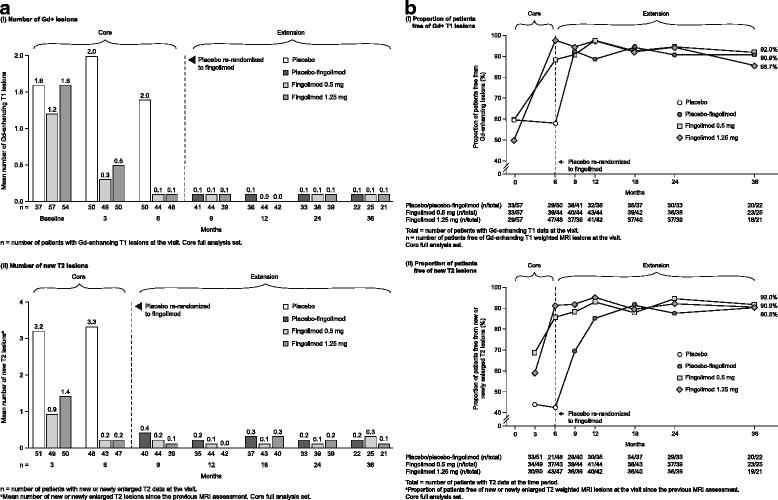
**a** Number of (*i*) Gd + lesions and (*ii*) new T2 lesions, and **b** proportion of patients free of (*i*) Gd + T1 and (*ii*) new T2 lesions in the core and extension study

The high proportion of patients reported free from Gd-enhanced T1 or new or newly enlarged T2 lesions from 6 months in those receiving continuous treatment [6], and from 12 months in those switching to fingolimod [7], was maintained during the 3-year extension and ranged from 85.7–92.0 to 90.5–92.0%, respectively, at month 36 (ranges provided are across all treatment groups and assessment time points during the extension; Fig. 2). Similarly, the reductions in mean numbers of Gd-enhanced or new or newly enlarged T2 lesions achieved at 12 months in both continuous and switch groups [7] ranged from 0.0–0.1 to 0.0–0.3, respectively, during the 3-year extension (Fig. 2).

**Fig. 3:**
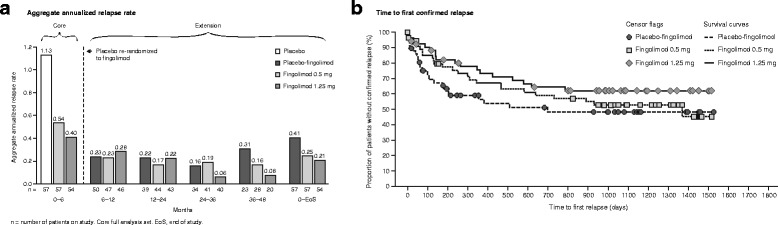
Relapse outcomes presented as **a** aggregate annualized relapse rate up to end of study by time period and treatment, and **b** Kaplan-Meier plot of time to first confirmed relapse

Continuous fingolimod 0.5 or 1.25 mg treatment during the extension phase resulted in reductions in ARR compared with the core phase (Fig. 3a). A reduction in ARR was observed at months 6–12 (fingolimod 0.5 mg, from 0.539 to 0.227 [57.9% reduction]; fingolimod 1.25 mg, from 0.404 to 0.284 [29.7% reduction]; Fig. 3a); the reduction in both groups was sustained to the end of study (month 0–end of study [EoS]: fingolimod 0.5 mg, 0.247; fingolimod 1.25 mg, 0.208; Fig. 3a). After switching to active treatment, a 79.5% decrease in ARR was observed in the placebo-fingolimod group in the first 6 months of the extension (from 1.131 to 0.232). Overall, 45–62% of patients in the continuous fingolimod groups and 48% of patients in the placebo-fingolimod group were relapse-free at the end of study based on Kaplan-Meier estimates (Fig. 3b).

Among patients treated continuously with fingolimod, disability levels remained stable in the extension study (month 6 vs EoS: fingolimod 0.5 mg, mean EDSS score = 2.30 vs 2.24; fingolimod 1.25 mg, mean EDSS score = 1.70 vs 1.54). Among patients who switched from placebo to fingolimod, disability levels were lower in the extension study compared with the core phase (month 6 vs EoS: mean EDSS score = 2.20 vs 1.80). In addition, 74.3% (fingolimod 0.5 mg) and 90.6% (placebo-fingolimod) of patients were free from 3 month confirmed disability progression and 87.1% (fingolimod 0.5 mg) and 92.3% (placebo-fingolimod) of patients were free from 6 month confirmed disability progression at last EDSS assessment.

### Safety and tolerability

The majority of AEs were mild or moderate in severity. SAEs were reported in 19 (13.3%) patients during the extension study, with the range in the continuous fingolimod and placebo-fingolimod switch groups (3.7–21.7%) being similar to that reported in the core study for the placebo and fingolimod groups (5.3–20.4%) [6]. During the extension, bradycardia was the only SAE reported in more than one patient (placebo-fingolimod 1.25 mg, *n* = 2 [8.7%]). One case of macular edema was reported as an SAE in a patient treated continuously with fingolimod 0.5 mg for 27 months, although medical intervention was not required to resolve the event and the case was subsequently unconfirmed on central review by a retina specialist from the data and safety monitoring board. Infections and infestations classified as SAEs were reported in two patients treated in the continuous fingolimod 1.25 mg group (appendicitis, *n* = 1; urinary tract infection, *n* = 1). During the extension study, SAEs leading to study drug discontinuation were reported for five patients (3.5%): three in the continuous fingolimod 0.5 mg group (6.4%; breast cancer, central nervous system lymphoma, MS relapse), one in the placebo-fingolimod 1.25 mg group (NMO relapse as leukoencephalopathy) and one in the placebo-fingolimod 0.5 mg group (NMO relapse). NMO relapse SAEs occurred in two patients who were seropositive for anti-AQP4 antibodies; these were both previously described in [7].

The proportion of patients with AEs during the extension study was similar across groups (Table 2) and similar to the 6-month core study [6]. Nasopharyngitis was the most common AE (58.7%), followed by abnormal liver function test (21.0%), lymphopenia (13.3%), stomatitis (10.5%) and headache (10.5%; Table 2). Over the course of the extension study, infections and infestations were reported in 114 patients (79.7%); the majority were categorized as mild or moderate. Herpes zoster infections were reported in five patients; two in the placebo-fingolimod 1.25 mg group, one in the placebo-fingolimod 0.5 mg group, one in the fingolimod 1.25 mg group and one in the fingolimod 0.5 mg group. Influenza occurred in 14 patients, and was more frequent in the continuous and placebo-fingolimod 1.25 mg switch groups (Table 2). There were no reports of infections and infestations AEs leading to study drug discontinuation during the extension study. Cardiac AEs were reported in 11 patients (7.7%), and all of these events occurred following initiation of fingolimod in patients who switched from placebo. Overall, 22 patients out of 143 (15.4%) discontinued study drug due to AEs. AEs leading to permanent study drug discontinuation were reported more frequently in patients who switched from placebo to fingolimod during the extension phase (21.7–29.6%) than in continuous fingolimod groups (4.3–14.9%). The most frequent causes of discontinuation, which were reported in at least two patients, included leukopenia (2.8%), abnormal liver function test (2.1%), hepatic enzyme increase (1.4%) and decreased lymphocyte count (1.4%).

**Table 2:** Adverse events and first-dose monitoring events during the extension study

	Placebo-fingolimod 0.5 mg (*n* = 27)	Placebo-fingolimod 1.25 mg (*n* = 23)	Fingolimod 0.5 mg (*n* = 47)	Fingolimod 1.25 mg (*n* = 46)	Total (*n* = 143)
Any AE	27 (100)	23 (100)	45 (95.7)	44 (95.7)	139 (97.2)
AEs leading to study drug discontinuation^a^	8 (29.6)	5 (21.7)	7 (14.9)	2 (4.3)	22 (15.4)
SAEs	1 (3.7)	5 (21.7)	8 (17.0)	5 (10.9)	19 (13.3)
SAEs leading to study drug discontinuation^b^	1 (3.7)	1 (4.3)	3 (6.4)	0 (0.0)	5 (3.5)
Frequent or special-interest adverse events					
Infections and infestations	22 (81.5)	17 (73.9)	37 (78.7)	38 (82.6)	114 (79.7)
Total upper respiratory tract infections	17 (63.0)	12 (52.2)	31 (66.0)	29 (63.0)	89 (62.2)
Nasopharyngitis	15 (55.6)	12 (52.2)	29 (61.7)	28 (60.9)	84 (58.7)
Pharyngitis	1 (3.7)	2 (8.7)	5 (10.6)	4 (8.7)	12 (8.4)
Upper respiratory tract infection	1 (3.7)	0 (0.0)	2 (4.3)	1 (2.2)	4 (2.8)
Influenza viral infections	2 (7.4)	3 (13.0)	3 (6.4)	6 (13.0)	14 (9.8)
Lower respiratory tract and lung infections	1 (3.7)	0 (0.0)	1 (2.1)	2 (4.3)	4 (2.8)
Bronchitis	1 (3.7)	0 (0.0)	1 (2.1)	2 (4.3)	4 (2.8)
Herpes viral infections	2 (7.4)	4 (17.4)	1 (2.1)	3 (6.5)	10 (7.0)
Herpes zoster	1 (3.7)	2 (8.7)	1 (2.1)	1 (2.2)	5 (3.5)
Oral herpes	0 (0.0)	2 (8.7)	0 (0.0)	0 (0.0)	2 (1.4)
Urinary tract infections	2 (7.4)	1 (4.3)	4 (8.5)	9 (19.6)	16 (11.2)
Cystitis	2 (7.4)	1 (4.3)	3 (6.4)	7 (15.2)	13 (9.1)
Vascular disorders	1 (3.7)	1 (4.3)	4 (8.5)	2 (4.3)	8 (5.6)
Hypertension	0 (0.0)	1 (4.3)	3 (6.4)	1 (2.2)	5 (3.5)
Eye disorders	5 (18.5)	3 (13.0)	14 (29.8)	9 (19.6)	31 (21.7)
Macular edema	0 (0.0)	0 (0.0)	1 (2.1) ^c^	0 (0.0)	1 (0.7)
Nervous system disorders	4 (14.8)	6 (26.1)	14 (29.8)	9 (19.6)	33 (23.1)
Headache	1 (3.7)	3 (13.0)	8 (17.0)	3 (6.5)	15 (10.5)
Investigations	13 (48.1)	14 (60.9)	15 (31.9)	17 (37.0)	59 (41.3)
Abnormal liver function test	7 (25.9)	9 (39.1)	6 (12.8)	8 (17.4)	30 (21.0)
Alanine aminotransferase increased	2 (7.4)	0 (0.0)	0 (0.0)	1 (2.2)	3 (2.1)
Gamma-glutamyl transferase increased	1 (3.7)	2 (8.7)	1 (2.1)	0 (0.0)	4 (2.8)
Aspartate amino-transferase increased	1 (3.7)	0 (0.0)	0 (0.0)	1 (2.2)	2 (1.4)
Hepatic enzyme increased	1 (3.7)	1 (4.3)	1 (2.1)	0 (0.0)	3 (2.1)
Lymphocyte count decreased	3 (11.1)	2 (8.7)	3 (6.4)	2 (4.3)	10 (7.0)
Gastrointestinal disorders	11 (40.7)	6 (26.1)	22 (46.8)	23 (50.0)	62 (43.4)
Stomatitis	4 (14.8)	1 (4.3)	4 (8.5)	6 (13.0)	15 (10.5)
Diarrhea	1 (3.7)	2 (8.7)	3 (6.4)	6 (13.0)	12 (8.4)
Constipation	1 (3.7)	0 (0.0)	5 (10.6)	2 (4.3)	8 (5.6)
Skin and subcutaneous tissue disorders	8 (29.6)	9 (39.1)	14 (29.8)	16 (34.8)	47 (32.9)
Rash	3 (11.1)	4 (17.4)	1 (2.1)	0 (0.0)	8 (5.6)
Blood and lymphatic system disorders	7 (25.9)	5 (21.7)	10 (21.3)	13 (28.3)	35 (24.5)
Lymphopenia	2 (7.4)	3 (13.0)	6 (12.8)	8 (17.4)	19 (13.3)
Leukopenia	4 (14.8)	1 (4.3)	2 (4.3)	5 (10.9)	12 (8.4)
Respiratory, thoracic and mediastinal disorders	1 (3.7)	4 (17.4)	6 (12.8)	9 (19.6)	20 (14.0)
Metabolism and nutrition disorders	4 (14.8)	1 (4.3)	4 (8.5)	7 (15.2)	16 (11.2)
Hyperlipidemia	2 (7.4)	0 (0.0)	2 (4.3)	5 (10.9)	9 (6.3)
Psychiatric disorders	2 (7.4)	2 (8.7)	8 (17.0)	4 (8.7)	16 (11.2)
Neoplasms benign, malignant and unspecified (including cysts and polyps)	0 (0.0)	1 (4.3)	9 (19.1)	1 (2.2)	11 (7.7)
Skin papilloma	0 (0.0)	0 (0.0)	6 (12.8)	0 (0.0)	6 (4.2)
First-dose monitoring events^d^					
Cardiac disorders	4 (14.8)	7 (30.4)	0 (0.0)	0 (0.0)	11 (7.7)
Atrioventricular block second degree	0 (0.0)	3 (13.0)	0 (0.0)	0 (0.0)	3 (2.1)
Bradycardia	0 (0.0)	3 (13.0)	0 (0.0)	0 (0.0)	3 (2.1)
Serious adverse events^e^					
Infections and infestations	0 (0.0)	0 (0.0)	0 (0.0)	2 (4.3)	2 (1.4)
Appendicitis	0 (0.0)	0 (0.0)	0 (0.0)	1 (2.2)	1 (0.7)
Urinary tract infection	0 (0.0)	0 (0.0)	0 (0.0)	1 (2.2)	1 (0.7)
Cardiac disorders	0 (0.0)	2 (8.7)	0 (0.0)	0 (0.0)	2 (1.4)
Bradycardia	0 (0.0)	2 (8.7)	0 (0.0)	0 (0.0)	2 (1.4)
Neoplasms benign, malignant and unspecified (including cysts and polyps)	0 (0.0)	0 (0.0)	2 (4.3)	0 (0.0)	2 (1.4)
Breast cancer	0 (0.0)	0 (0.0)	1 (2.1)	0 (0.0)	1 (0.7)
CNS lymphoma	0 (0.0)	0 (0.0)	1 (2.1)	0 (0.0)	1 (0.7)
Nervous system disorders	1 (3.7)	2 (8.7)	3 (6.4)	1 (2.2)	7 (4.9)
Status epilepticus	0 (0.0)	0 (0.0)	0 (0.0)	1 (2.2)	1 (0.7)
Convulsion	0 (0.0)	0 (0.0)	1 (2.1)	0 (0.0)	1 (0.7)
Leukoencephalopathy	0 (0.0)	1 (4.3)	0 (0.0)	0 (0.0)	1 (0.7)
Multiple sclerosis relapse	0 (0.0)	0 (0.0)	1 (2.1)	0 (0.0)	1 (0.7)
Myoclonus	0 (0.0)	0 (0.0)	1 (2.1)	0 (0.0)	1 (0.7)
Neuromyelitis optica	1 (3.7)	0 (0.0)	0 (0.0)	0 (0.0)	1 (0.7)
Radiculitis	0 (0.0)	1 (4.3)	0 (0.0)	0 (0.0)	1 (0.7)

Data are patients (*n* [%]) in whom events were reported by at least 10% of patients in one or more treatment group, or were events of special interest. Adverse events listed are classified as system organ class (e.g. infections) followed by high-level term (e.g. total upper respiratory tract infections) and/or preferred term (e.g. upper respiratory tract infection, nasopharyngitis, sinusitis) for the event, as applicable

*Abbreviations*: *AE* Adverse event, *AQP4* Aquaporin-4, *CNS* Central nervous system, FDM, first dose monitoring, *SAE* Serious adverse event

^a^Any AE leading to study drug discontinuation includes any patient whose primary or secondary reason for discontinuing the study drug was an AE

^b^SAEs leading to study drug discontinuation were neoplasms (breast cancer and CNS lymphoma), and nervous system disorders (MS relapse, leukoencephalopathy and neuromyelitis optica). Leukoencephalopathy and neuromyelitis optica were reported in patients who were anti-AQP4 antibody-positive

^c^Not confirmed on central review by a retina specialist from the data and safety monitoring board

^d^FDM events for placebo-fingolimod switch groups on entry to the extension study

^e^SAE data are *n* (%) for events reported by at least two patients in one or more treatment group, unless otherwise stated

During the extension study, abnormal liver function test AEs were reported more frequently in the groups that switched from placebo to fingolimod (25.9–39.1%) than in the continuous fingolimod groups (12.8–17.4%; Table 2). In the continuous fingolimod groups, the frequency of abnormal liver function test AEs was lower during the extension study (12.8–17.4%) than during the core study (21.1–33.3%) [6]. Greater mean levels of alanine aminotransferase (ALT), aspartate aminotransferase (AST) and gamma-glutamyl transferase (GGT) were observed at month 6 in patients treated continuously with fingolimod than in patients receiving placebo, but these levels remained stable during the extension (Additional file 1: Table S2). After 15 days of fingolimod therapy in the extension (month 6.5), patients who had switched from placebo had increased levels of ALT, AST and GGT, and these were similar to the levels seen among patients treated with fingolimod during the core study. During months 18–36, levels of ALT, AST and GGT were similar across all patient groups (Additional file 1: Table S2). During the core study, the proportions of patients whose ALT and GGT levels were more than three-times the upper limit of normal (3 × ULN) were greater in those receiving fingolimod than in those receiving placebo; in the extension, the proportions of patients with ALT and GGT levels more than 3 × ULN were similar in the continuous and switch groups (Additional file 1: Table S3).

**Fig. 4:**
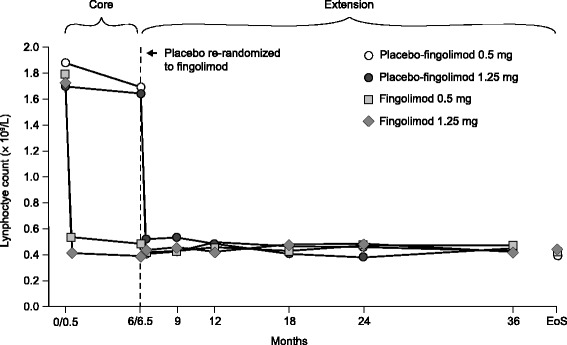
Change in mean lymphocyte count during the core and extension study (extension safety population). *EoS* (end of study): in this instance is the last non-missing value up to 2 days after the last dose date and is summarized as last assessment on study drug. Data at 0.5 months (day 15) for fingolimod 0.5 mg (*n* = 54) and 1.25 mg (*n* = 56) represent values obtained during the core study (safety population)

Lymphocyte counts in the continuous fingolimod 1.25 and 0.5 mg groups reduced to 22.1 and 26.9% of the core baseline levels at month 6 (Fig. 4). Subsequently, lymphocyte counts remained stable during the extension (28.3 and 26.3% of core baseline values, respectively). In patients who switched from placebo to fingolimod 1.25 or 0.5 mg, lymphocyte numbers reduced to 22.0 and 30.4% of core baseline levels at month 6.5, and remained stable thereafter (Fig. 4). At the end of study, the majority of patients in each group had lymphocyte counts greater than 0.20 × 10^9^/L, the threshold at which fingolimod treatment was interrupted in clinical studies (fingolimod 0.5 mg, 72.3%; fingolimod 1.25 mg, 71.7%; placebo-fingolimod 0.5 mg, 81.5%; placebo-fingolimod 1.25 mg, 69.6%). Absolute neutrophil and white blood cell (WBC) counts were reduced to a similar extent in the continuous and switch groups at study end, however no patient group had a neutrophil count < 1.0 × 10^9^/L at any stage in the study and all patient groups had WBC counts that were either normal or between 1.5–2.0 × 10^9^/L (Additional file 1: Table S4).

Throughout the core and extension study, no skin malignancy was detected on dermatological examination. Neoplasms were reported in 11 patients (7.7%). Breast cancer and central nervous system lymphoma were each reported in one patient on continuous fingolimod 0.5 mg. All other cases were reported as benign skin neoplasms (skin papilloma, *n* = 6; melanocytic naevus, *n* = 2; anogenital warts, *n* = 1).

No deaths occurred during the extension study for up to 3 months after study drug discontinuation. A 42-year-old man died approximately 1 year after study drug discontinuation (9 months on fingolimod 0.5 mg); cause of death was Epstein-Barr virus (EBV)-positive diffuse large B-cell lymphoma of the brain, skin, lungs, kidneys and thyroid gland; no MS pathology was detected in this case [7]. A second case of diffuse large B-cell lymphoma was reported in a 38-year old woman who received continuous fingolimod 0.5 mg treatment, with a suspected relationship to the study drug medication (onset 1250 days on fingolimod 0.5 mg). First symptoms of malignant lymphoma, identified retrospectively, were reported on 22 September 2011, 1155 days after the start of study medication. Following a computerized tomography scan of the head, which revealed aggravation of hydrocephalus, the patient was withdrawn from the study and the study drug medication was stopped (26 December 2011; 1250 days after the start of study medication). Following draining for hydrocephalus, B-cell lymphoma was confirmed on biopsy analysis (12 January 2012), and chemotherapy was started. Subsequently, multiple lesions previously observed on contrast-enhanced head MRI scan, and sites of aggregation in locations such as the right iliac bone, disappeared and the patient was discharged. During the extension, four patients receiving fingolimod (1.25 mg, *n* = 3; 0.5 mg, *n* = 1) became pregnant and therefore treatment was discontinued immediately. Two of these patients chose to terminate their pregnancies, while the remaining two patients gave birth to infants who were considered healthy. One patient receiving placebo terminated pregnancy during the core study.

## Discussion

In this 3-year extension of the original 6-month core study in Japanese patients with MS, fingolimod sustained low levels of disease activity and no new safety signals were observed [6, 7]. These results are important given that genetic, metabolic, lifestyle, incidence and clinical presentation differences exist between Japanese patients with MS and individuals with MS in Western countries [6, 9]. The current study was designed to exclude patients with NMO on the basis of spinal MRI criteria at screening [7], although anti-AQP4-antibody status (a more specific marker for NMO) was not used, hence it is possible that not all cases of NMO were excluded. Notably, results in a small number of patients tested retrospectively for anti-AQP4 antibodies suggested a lack of benefit of fingolimod treatment in patients who tested positive for anti-AQP4 antibodies [7]. However, the overall efficacy and safety findings of fingolimod treatment in this population of Japanese patients with MS were similar to those seen in predominantly Caucasian populations in the original phase 3 pivotal studies and their extensions [2, 4, 5, 9, 10].

A low level of disease activity, based on all efficacy endpoints (ARR, EDSS and MRI outcomes) observed at the end of the core study were sustained until the end of the extension study in patients treated with continuous fingolimod. Reduced MRI and relapse activity were observed within the first 6 months in patients who switched to fingolimod at the start of the extension study, and this was sustained until the end of the study. These findings are similar to those from the 6-month extension study [7], in which continuous fingolimod therapy for 12 months was associated with sustained reductions in relapse rates and MRI lesion activity compared with those in the core study. The current results suggest that continuous treatment with fingolimod provides long-term benefit to the patient.

Fingolimod was generally well tolerated and the safety profile was consistent with results from previous studies [2–5, 10], including those reported in the core and 6-month extension [6, 7]. This was reflected in the high patient retention rate (74.8%) at the end of the 3-year study extension. The incidences of infections and cardiac AEs were low. A case of possible macular edema was unconfirmed and resolved without treatment. A single case of EBV-positive B-cell lymphoma was reported in this extension. One patient died approximately 1 year after study drug discontinuation (0.5 mg fingolimod) due to EBV-positive diffuse large B-cell lymphoma but without any MS pathology, suggesting the existence of brain lymphoma before commencing fingolimod medication. Overall, the incidence of malignancy was low and similar to that in other fingolimod studies [11]. Expected elevations in liver function test enzymes and lymphopenia occurred shortly after initiation of fingolimod therapy, and levels remained stable for the duration of the extension [2, 4, 5]. There was a single case of NMO relapse reported as a SAE during months 7–12 of the extension in one of four AQP4 antibody-positive patients who experienced relapses during the entire study; this case has been discussed previously [7]. NMO exacerbation has been reported in patients treated with MS disease-modifying therapies who were subsequently found to be anti-AQP4 antibody-positive [12], and in whom the initial diagnosis of MS was changed to NMO [13]. As the nature of relapses in all anti-AQP4-positive patients seen after commencing fingolimod appeared to be similar to previous relapses, it is conceivable that fingolimod did not alter but rather enhanced disease activity through an unknown mechanism [7].

In addition to the small sample size, a consequence of the recruitment challenge associated with the low incidence of MS in Japan, a drawback of this extension study that limits conclusions regarding efficacy is the lack of a placebo-control group, and the reduction in comparator groups following the need for all patients receiving fingolimod 1.25 mg to switch to fingolimod 0.5 mg. Nevertheless, continuous fingolimod treatment over 36 months was associated with maintained efficacy and a manageable safety profile.

## Conclusion

These results indicate that there is a long-term benefit associated with treating Japanese patients with relapsing MS continuously with fingolimod.

## Additional file

Additional file 1: Table S1. Duration of exposure to fingolimod in patients entering the extension study. **Table S2.** Plasma levels of liver enzymes at core study baseline and in the extension study. **Table S3.** Proportion of patients with normal and elevated levels of liver enzymes. **Table S4.** Proportion of patients with normal and decreased levels of hematological parameters. (DOCX 35 kb)

## Abbreviations

AE: Adverse event
ALT: Alanine aminotransferase
AQP4: Aquaporin-4
ARR: Annualized relapse rate
AST: Aspartate aminotransferase
BMI: Body mass index
EBV: Epstein-Barr virus
EDSS: Expanded Disability Status Scale
EoS: End of study
FAS: Full analysis set
Gd: Gadolinium
GGT: Gamma-glutamyl transferase
IFN: Interferon
MRI: Magnetic resonance imaging
MS: Multiple sclerosis
NMO: Neuromyelitis optica
RRMS: Relapsing˗̶remitting multiple sclerosis
SAE: Serious adverse event
SD: Standard deviation
SOC: System organ class
ULN: Upper limit of normal
WBC: White blood cell
