# Supplementary material for: Long-term efficacy and safety of fingolimod in Japanese patients with relapsing multiple sclerosis: 3-year results of the phase 2 extension study
Source: BMC Neurol. 2017 Jan 28;17:17. doi: 10.1186/s12883-017-0794-5 (PMC5273805; doi:10.1186/s12883-017-0794-5)
Supplement: Additional file 1: Table S1. — Duration of exposure to fingolimod in patients entering the extension study. Table S2. Plasma levels of liver enzymes at core study baseline and in the extension study. Table S3. Proportion of patients with normal and elevated levels of liver enzymes. Table S4. Proportion of patients with normal and decreased levels of hematological parameters. (DOCX 35 kb) [file 12883_2017_794_MOESM1_ESM.docx]

**Additional file**

**Table S1 Duration of exposure to fingolimod in patients entering the extension study**

|  | **Placebo-fingolimod 0.5 mg**  **(n = 27)** | **Placebo-fingolimod**  **1.25 mg**  **(n = 23)** | **Fingolimod**  **0.5 mg**  **(n = 47)** | **Fingolimod 1.25 mg**  **(n = 46)** |
| --- | --- | --- | --- | --- |
| Exposure (days) |  |  |  |  |
| Mean (SD) | 734.3 (435.05) | 705.9 (455.63) | 1093.0 (336.96) | 1020.6 (375.79) |
| Median | 857 | 820 | 1180 | 1070 |
| Range | 14–1345 | 14–1276 | 215–1535 | 121–1518 |
| Duration of exposure (days) n, % |  |  |  |  |
| ≥ 360 | 20 (74.1) | 15 (65.2) | 44 (93.6) | 41 (89.1) |
| ≥ 720 | 19 (70.4) | 15 (65.2) | 40 (85.1) | 39 (84.8) |
| ≥ 1080 | 7 (25.9) | 6 (26.1) | 27 (57.4) | 20 (43.5) |

In the core study, all patients in the continuous 1.25 mg and placebo-fingolimod 1.25 mg groups were switched to fingolimod 0.5 mg by 22 February 2010. Before the switch to fingolimod 0.5 mg, the percentages of patients who experienced ≥ 360 days and ≥ 720 days of exposure to fingolimod 1.25 mg were 45.7% and 13.0%, respectively, in the continuous fingolimod 1.25 mg group, while the percentages were 26.1% and 0.0%, respectively, in the placebo-fingolimod 1.25 mg switch group.

Abbreviations: *SD* Standard deviation.

**Table S2 Plasma levels of liver enzymes at core study baseline and in the extension study**

|  | **Placebo-fingolimod 0.5 mg**  **(n = 27)** | **Placebo-fingolimod**  **1.25 mg**  **(n = 23)** | **Fingolimod**  **0.5 mg**  **(n = 47)** | **Fingolimod 1.25 mg**  **(n = 46)** |
| --- | --- | --- | --- | --- |
| **AST (U/L), mean (SD)** |  |  |  |  |
| Core baseline | 18.3 (4.9) | 20.4 (6.6) | 17.6 (5.2) | 17.8 (5.4) |
| Month 6 | 21.0 (11.0) | 22.0 (11.0) | 24.6 (10.4) | 26.5 (11.5) |
| Month 6.5 | 25.5 (17.0) | 27.4 (20.2) | 23.9 (8.8) | 27.4 (11.1) |
| Month 9 | 24.9 (10.9) | 36.4 (21.5) | 25.4 (11.2) | 25.5 (10.4) |
| Month 12 | 23.8 (9.7) | 35.2 (25.4) | 23.4 (8.4) | 28.0 (16.5) |
| Month 18 | 25.6 (11.1) | 26.2 (8.9) | 26.4 (16.8) | 24.8 (9.4) |
| Month 24 | 22.0 (5.7) | 23.6 (7.0) | 22.1 (7.7) | 25.9 (10.0) |
| Month 36 | 23.8 (7.1) | 22.5 (6.2) | 22.4 (5.5) | 25.7 (8.8) |
| EoS | 27.7 (19.6) | 31.3 (21.7) | 23.5 (7.4) | 23.5 (9.8) |
| **ALT (U/L), mean (SD)** |  |  |  |  |
| Core baseline | 15.0 (6.1) | 19.3 (10.5) | 15.9 (8.4) | 16.1 (8.0) |
| Month 6 | 18.8 (13.4) | 20.4 (13.0) | 30.9 (22.1) | 37.4 (29.8) |
| Month 6.5 | 30.9 (30.4) | 28.4 (22.7) | 30.4 (19.8) | 38.2 (26.5) |
| Month 9 | 29.6 (23.2) | 45.2 (28.0) | 35.1 (26.4) | 34.4 (22.6) |
| Month 12 | 31.9 (23.4) | 46.3 (47.5) | 30.0 (18.6) | 38.9 (29.2) |
| Month 18 | 36.6 (27.0) | 31.3 (19.6) | 32.6 (22.0) | 34.5 (24.9) |
| Month 24 | 25.8 (11.4) | 25.6 (10.1) | 27.7 (15.7) | 32.9 (19.2) |
| Month 36 | 30.7 (20.0) | 23.6 (8.1) | 29.4 (15.1) | 28.9 (11.2) |
| EoS | 36.0 (26.9) | 40.9 (48.3) | 31.8 (18.7) | 33.1 (26.1) |
| **GGT (U/L), mean (SD)** |  |  |  |  |
| Core baseline | 18.2 (8.5) | 31.9 (38.3) | 20.2 (10.9) | 22.6 (20.9) |
| Month 6 | 21.7 (12.8) | 38.0 (64.0) | 63.1 (62.8) | 78.1 (94.8) |
| Month 6.5 | 32.6 (37.9) | 52.2 (69.7) | 59.9 (56.5) | 90.1 (122.1) |
| Month 9 | 39.0 (30.8) | 125.5 (173.3) | 71.4 (66.1) | 76.5 (75.5) |
| Month 12 | 47.1 (56.3) | 106.1 (111.0) | 72.7 (71.5) | 77.1 (70.0) |
| Month 18 | 59.1 (52.0) | 106.4 (168.4) | 83.2 (83.4) | 75.2 (55.3) |
| Month 24 | 58.1 (61.8) | 83.4 (77.1) | 76.2 (77.8) | 71.7 (42.9) |
| Month 36 | 41.4 (20.2) | 82.5 (84.4) | 76.1 (57.1) | 81.1 (52.4) |
| EoS | 76.2 (96.7) | 107.5 (154.8) | 70.6 (64.1) | 76.4 (95.2) |

*EoS* end of study, in this instance, the last non-missing value up to 2 days after last dose date and is summarized as last assessment on study drug.

Abbreviations: *ALT* Alanine aminotransferase; *AST* Aspartate aminotransferase; *GGT* Gamma-glutamyl-transferase; *SD* Standard deviation; *U/L* Units/litre.

**Table S3 Proportion of patients with normal and elevated levels of liver enzymes**

|  | **Months 0–6 (core)** | | |  | **Month 6–EoS (extension)^a^** | | | |
| --- | --- | --- | --- | --- | --- | --- | --- | --- |
|  | **Placebo** | **Fingolimod**  **0.5 mg** | **Fingolimod 1.25 mg** |  | **Placebo-fingolimod**  **0.5 mg** | **Placebo-fingolimod 1.25 mg** | **Fingolimod**  **0.5 mg** | **Fingolimod 1.25 mg** |
|  | **(n = 57)** | **(n = 57)** | **(n = 54)** |  | **(n = 27)** | **(n = 23)** | **(n = 47)** | **(n = 46)** |
| **AST** | | | | | | | | |
| No abnormality | 50 (87.7) | 45 (80.4) | 35 (64.8) |  | 14 (51.9) | 13 (56.5) | 31 (66.0) | 29 (63.0) |
| > ULN | 7 (12.3) | 11 (19.6) | 19 (35.2) |  | 13 (48.1) | 10 (43.5) | 16 (34.0) | 17 (37.0) |
| ≥ 2 x ULN | 2 (3.5) | 2 (3.6) | 3 (5.6) |  | 3 (11.1) | 3 (13.0) | 2 (4.3) | 3 (6.5) |
| ≥ 3 x ULN | 1 (1.8) | 1 (1.8) | 0 (0.0) |  | 0 (0.0) | 2 (8.7) | 1 (2.1) | 0 (0.0) |
| ≥ 5 x ULN | 0 (0.0) | 0 (0.0) | 0 (0.0) |  | 0 (0.0) | 0 (0.0) | 0 (0.0) | 0 (0.0) |
| ≥ 10 x ULN | 0 (0.0) | 0 (0.0) | 0 (0.0) |  | 0 (0.0) | 0 (0.0) | 0 (0.0) | 0 (0.0) |
| ≥ 20 x ULN | 0 (0.0) | 0 (0.0) | 0 (0.0) |  | 0 (0.0) | 0 (0.0) | 0 (0.0) | 0 (0.0) |
| **ALT** | | | | | | | | |
| No abnormality | 48 (84.2) | 38 (67.9) | 26 (48.1) |  | 9 (33.3) | 10 (43.5) | 19 (40.4) | 18 (39.1) |
| > ULN | 9 (15.8) | 18 (32.1) | 28 (51.9) |  | 18 (66.7) | 13 (56.5) | 28 (59.6) | 28 (60.9) |
| ≥ 2 x ULN | 4 (7.0) | 8 (14.3) | 16 (29.6) |  | 10 (37.0) | 5 (21.7) | 14 (29.8) | 12 (26.1) |
| ≥ 3 x ULN | 2 (3.5) | 4 (7.1) | 7 (13.0) |  | 6 (22.2) | 2 (8.7) | 2 (4.3) | 7 (15.2) |
| ≥ 5 x ULN | 2 (3.5) | 0 (0.0) | 1 (1.9) |  | 0 (0.0) | 2 (8.7) | 0 (0.0) | 0 (0.0) |
| ≥ 10 x ULN | 0 (0.0) | 0 (0.0) | 0 (0.0) |  | 0 (0.0) | 0 (0.0) | 0 (0.0) | 0 (0.0) |
| ≥ 20 x ULN | 0 (0.0) | 0 (0.0) | 0 (0.0) |  | 0 (0.0) | 0 (0.0) | 0 (0.0) | 0 (0.0) |
| **GGT** | | | | | | | | |
| No abnormality | 46 (80.7) | 31 (55.4) | 17 (31.5) |  | 9 (33.3) | 8 (34.8) | 11 (23.4) | 7 (15.2) |
| > ULN | 11 (19.3) | 25 (44.6) | 37 (68.5) |  | 18 (66.7) | 15 (65.2) | 36 (76.6) | 39 (84.8) |
| ≥ 2 x ULN | 3 (5.3) | 13 (23.2) | 16 (29.6) |  | 10 (37.0) | 11 (47.8) | 25 (53.2) | 28 (60.9) |
| ≥ 3 x ULN | 1 (1.8) | 10 (17.9) | 11 (20.4) |  | 7 (25.9) | 8 (34.8) | 14 (29.8) | 16 (34.8) |
| ≥ 5 x ULN | 1 (1.8) | 6 (10.7) | 7 (13.0) |  | 4 (14.8) | 4 (17.4) | 7 (14.9) | 6 (13.0) |
| ≥ 10 x ULN | 0 (0.0) | 1 (1.8) | 1 (1.9) |  | 1 (3.7) | 3 (13.0) | 2 (4.3) | 1 (2.2) |
| ≥ 20 x ULN | 0 (0.0) | 0 (0.0) | 0 (0.0) |  | 0 (0.0) | 0 (0.0) | 0 (0.0) | 0 (0.0) |

Data are presented as n (%).

^a^Note that the treatment duration of the extension phase (month 6–end of study) was much longer than that of the core phase (month 0–6).

Abbreviations: *ALT* Alanine aminotransferase; *AST* Aspartate aminotransferase; *EoS* end of study; *GGT* Gamma-glutamyl-transferase; *ULN* Upper limit of normal.

**Table S4 Proportion of patients with normal and decreased levels of hematological parameters**

|  | | **Months 0–6 (core)** | | |  | **Month 6–end of study (extension)^a^** | | | |
| --- | --- | --- | --- | --- | --- | --- | --- | --- | --- |
|  | | **Placebo** | **Fingolimod**  **0.5 mg** | **Fingolimod 1.25 mg** |  | **Placebo-fingolimod**  **0.5 mg** | **Placebo-fingolimod 1.25 mg** | **Fingolimod**  **0.5 mg** | **Fingolimod 1.25 mg** |
|  | | **(n = 57)** | **(n = 57)** | **(n = 54)** |  | **(n = 27)** | **(n = 23)** | **(n = 47)** | **(n = 46)** |
| **Lymphocytes x 10^9^/L** | | | | | | | | | |
| < 0.8 | 5 (8.8) | | 53 (94.6) | 54 (100) |  | 26 (96.3) | 23 (100) | 47 (100) | 45 (97.8) |
| < 0.6 | 0 (0.0) | | 53 (94.6) | 53 (98.1) |  | 26 (96.3) | 22 (95.7) | 47 (100) | 44 (95.7) |
| < 0.4 | 0 (0.0) | | 35 (62.5) | 43 (79.6) |  | 23 (85.2) | 20 (87.0) | 42 (89.4) | 39 (84.8) |
| < 0.2 | 0 (0.0) | | 5 (8.9) | 11 (20.4) |  | 5 (18.5) | 7 (30.4) | 13 (27.7) | 13 (28.3) |
| < 0.1 | 0 (0.0) | | 0 (0.0) | 0 (0.0) |  | 0 (0.0) | 1 (4.3) | 0 (0.0) | 1 (2.2) |
| **Neutrophils x 10^9^/L** | | | | | | | | | |
| < 1.5 | 3 (5.3) | | 5 (8.9) | 9 (16.7) |  | 6 (22.2) | 3 (13.0) | 12 (25.5) | 14 (30.4) |
| < 1.0 | 1 (1.8) | | 0 (0.0) | 0 (0.0) |  | 0 (0.0) | 0 (0.0) | 0 (0.0) | 0 (0.0) |
| < 0.5 | 1 (1.8) | | 0 (0.0) | 0 (0.0) |  | 0 (0.0) | 0 (0.0) | 0 (0.0) | 0 (0.0) |
| **WBCs x 10^9^/L** | | | | | | | | | |
| > ULN | 0 (0.0) | | 0 (0.0) | 0 (0.0) |  | 0 (0.0) | 0 (0.0) | 0 (0.0) | 0 (0.0) |
| Normal | 50 (87.7) | | 15 (26.8) | 10 (18.5) |  | 4 (14.8) | 3 (13.0) | 6 (12.8) | 5 (10.9) |
| < 2.0 | 1 (1.8) | | 2 (3.6) | 3 (5.6) |  | 3 (11.1) | 1 (4.3) | 7 (14.9) | 9 (19.6) |
| < 1.5 | 0 (0.0) | | 0 (0.0) | 0 (0.0) |  | 0 (0.0) | 0 (0.0) | 0 (0.0) | 0 (0.0) |
| < 1.0 | 0 (0.0) | | 0 (0.0) | 0 (0.0) |  | 0 (0.0) | 0 (0.0) | 0 (0.0) | 0 (0.0) |

Data are presented as n (%).

^a^Note that the treatment duration of the extension phase (month 6–end of study) was much longer than that of the core phase (month 0–6).

Abbreviations: *ULN* Upper limit of normal; *WBC* White blood cell.
